# Ganoderma lingzhi (Reishi Mushroom)-Induced Acute Liver Injury in the Setting of Alcohol Use: A Case Report and Review of the Literature

**DOI:** 10.7759/cureus.45953

**Published:** 2023-09-25

**Authors:** Roupen Guedikian, Brandon Kim, Gagandeep Singh, Rebekah Alexander

**Affiliations:** 1 Internal Medicine, Arrowhead Regional Medical Center, Colton, USA; 2 Medicine, California University of Science and Medicine, Colton, USA; 3 Research, California University of Science and Medicine, Colton, USA

**Keywords:** toxin induced liver injury, mushroom toxicity, toxic hepatitis, reishi, ganoderma lucidum

## Abstract

As many prior case reports have shown, unregulated supplements and alcohol are both known to cause varying degrees of hepatotoxicity. We present a case of a 47-year-old male who presented to the hospital with headache and abdominal pain after consuming Reishi Mushroom (Ganoderma lingzhi) powder and alcohol. The patient was found to have acute hepatitis with significant transaminitis, which was managed conservatively with N-acetylcysteine and IV fluids. Two-week follow-up labs demonstrated complete resolution of the patient’s symptoms and laboratory abnormalities. Despite the growing popularity of mushroom-based supplements, limited research has been done on the systemic effects that can manifest with co-ingestion of other substances such as alcohol.

## Introduction

Dietary and herbal supplements have been known to cause liver injury. With over 100,000 dietary and herbal supplements available for purchase in stores throughout the United States and no federal regulations managing these products, they have been implicated in cases of hepatic failure. The incidence and mechanism of herbal-supplement-induced liver injury are still largely unknown; however, there has been a rise in the number of supplement-induced liver injury cases. Since herbal supplement-induced liver injury is uncommon, it is an often overlooked diagnosis in patients with transaminitis. One ingredient widely used in herbal supplements is Ganoderma lingzhi, also known as Lucidum or Reishi. G. lingzhi is a fungus that is used throughout Asia in traditional Eastern medicine touted to have many medicinal capabilities [1]. Although this fungus has been shown to have antioxidant and even hepatoprotective effects, in certain reported cases it has been associated with hepatotoxicity [2,3]. As herbal supplementals continue to be used worldwide, it is important to consider and appropriately recognize herbal supplement-induced liver injury in patients presenting with abnormal liver enzymes.

## Case presentation

A 47-year-old male with a past medical history of hypertension, chronic pain after inguinal hernia repair, and fatty liver disease, presented to the emergency department with nausea, vomiting, abdominal pain, and headache. The patient reported that his symptoms began three days prior, but he became more concerned when his blood pressure was measured in the 180s/130s, prompting him to visit the emergency room. Four days prior to the presentation, the patient had worsening of his chronic neuropathic pain, which extended from his abdomen to the right leg. The patient stated that to relieve the pain, over the next three days, he consumed two to three 750 mL bottles of vodka as well as multiple tablespoons of Reishi mushroom powder. On arrival at the emergency department, the patient was hypertensive with a blood pressure of 159/108. The remainder of the vital signs were within normal limits including temperature (97.7℉), heart rate (72), respiratory rate (18), and SpO_2_ (98% on room air). Physical exam was notable for diffuse abdominal tenderness; no jaundice was noted, and the patient was alert and oriented to person, place, and time.

Laboratory values were notable for elevated aspartate phosphatase (301), alanine transaminase (990), total bilirubin (1.5), and lipase (70). The baseline hepatic function panel nine months prior showed normal levels of aspartate phosphatase (10), alanine transaminase (13), and total bilirubin (0.4). Viral hepatitis panel and ethanol levels were negative. Ultrasound of the right upper quadrant visualized fatty infiltration of the liver; CT abdomen and pelvis with IV contrast was negative for any acute changes. The patient was given N-acetylcysteine and Lactated Ringer’s Solution at a rate of 100 mL/hr for a total of 1 liter. Repeat hepatic function panel the next day showed decreasing AST (110), ALT (612), and total bilirubin (1.2).

**Table 1:** Hepatic function panel mg = milligram, dL = deciliter, U = unit, L = liter, g = gram

Blood test ordered	Patient value on admission	Patient value on Day 2	Reference range
Total Bilirubin (mg/dL)	1.5	1.2	0.0 - 1.2
Bilirubin, Direct (mg/dL)	-	0.2	0 - 0.4
Alkaline Phosphatase (U/L)	84	70	35 - 125
AST (U/L)	301	110	5 - 40
ALT (U/L)	990	612	5 - 40
Total Protein (g/dL)	7	6.1	6.0 - 8.0
Albumin (g/dL)	4.5	3.8	3.5 - 4.9

**Figure 1:**
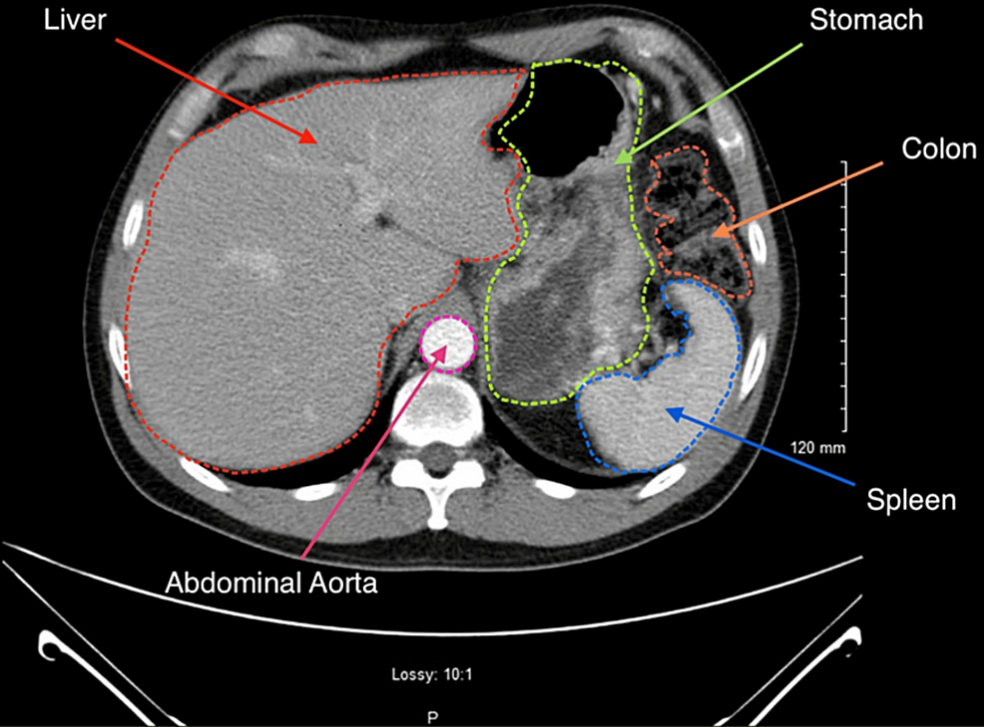
Computed tomography (CT) abdomen and pelvis with contrast The CT examination of the liver reveals an unremarkable appearance. The liver is of normal size and shape. There are no focal lesions, masses, or areas of abnormal enhancement noted within the hepatic parenchyma. The liver demonstrates homogeneous attenuation, and there are no signs of cirrhosis, fatty infiltration, or vascular abnormalities. The adjacent structures appear normal.

Due to improvement in liver function testing, the patient was discharged on day 2 of admission and advised to avoid consumption of Reishi mushroom and alcohol. On a two-week follow-up, the patient's abdominal pain, nausea, and headache had resolved. The comprehensive metabolic panel showed an ALT of 45 and AST of 16, confirming that the patient’s acute live injury/transaminitis had resolved.

## Discussion

G. lingzhi has been widely recognized across various cultures for thousands of years as a medicinal mushroom with prolific health benefits and has been promoted as a treatment for multiple conditions, such as cancer, heart disease, liver disease, and infection [4]. Research has postulated that these effects can be attributed to the antioxidant, anti-inflammatory, and immunomodulatory properties of the major polysaccharides found in the mushroom [5]. The mushroom is marketed in various forms as dietary supplements (e.g., capsule pills, ground powder, tea), primarily derived from intact fruiting bodies ground to powder form. The efficacy of these various forms remains a subject of controversy and is currently under significant scientific dispute. Wanmuang et al. described a case of fatal fulminant hepatitis after a patient switched from taking the traditionally boiled Lingzhi to ingesting Lingzhi powder for one to two months [6].

Here, we present another interesting case of a patient presenting with incidental hepatitis findings two days after ingesting Lingzhi mushroom powder with concomitant alcohol consumption. Despite Reishi demonstrating hepatoprotective properties, as evidenced by its effectiveness in mitigating alcohol-induced liver injury, our patient's significantly elevated liver enzymes and inconclusive investigation results point to the mushroom powder as the likely inducing factor of his hepatotoxicity aggravated by alcohol consumption in the setting of fatty liver disease [7]. The baseline hepatic function panel from nine months prior showed normal liver enzyme levels, indicating that the recent abnormalities were acute in nature. Furthermore, the absence of acute changes on the CT scan indicates that there were no other recent structural insults to the liver and rules out any alternative etiology of acute liver failure secondary to structural pathology. Of note, there were only abnormal ultrasound findings of fatty infiltration of the liver, which was at baseline. The patient's consumption of a significant amount of alcohol (3 x 750 mL bottles of vodka/whiskey over three days) likely played a role in his presentation. Nevertheless, the patient's transaminitis results differ from the typical pattern observed in alcoholic hepatitis, where an AST:ALT ratio greater than 1.5 or 2 would be expected. The combination of Reishi mushroom powder and ethanol may have synergistic effects on liver injury, potentially exacerbating the elevated liver enzymes and bilirubin observed. A recent study by Guo et al. showed high doses of ganoderic acids-rich G. lucidum ethanol (GLE) extract, a type of preparation also derived from fruiting bodies of the mushroom, significantly down-regulated mRNA levels of CYP2E1 in mice [8]. The CYP2E1 is an enzyme that is part of the principal pathway responsible for metabolizing ethanol. If the consumption of G. lingzhi decreases the concentration of CYP2E1, the metabolism of ethanol would be reduced, thus increasing the risk of ethanol-induced hepatitis.

## Conclusions

In this report, we have presented a case of a 47-year-old male who was found to have hepatitis, confirmed by significant increases in liver function tests. The patient's history and hospital workup revealed that the likely etiology of the hepatitis was toxic due to concomitant alcohol and mushroom consumption. Since ethanol is a common cause of acute hepatitis, the impact of the patient's G. lingzhi (Reishi Mushroom Powder) consumption was initially overlooked. However, as demonstrated in prior sections, G. lingzhi use has been shown to have toxic effects on the liver, with some case reports directly associating its use with episodes of fulminant hepatitis. Furthermore, a systematic review of the literature revealed that G. lingzhi has been shown to directly inhibit one of the key enzymes (CYP2E1) in the metabolic pathway of ethanol. Currently, marketing descriptions and instructions on the use of Reishi mushrooms do not consider any possible interactions with alcohol use. As the worldwide use of mushrooms and various other herbal supplements continues to increase, it is imperative that research into these compounds also expands appropriately. Interactions with commonly consumed and often abused substances such as ethanol must be considered and scrutinized in as much detail as possible.
